# dTAG-Mediated Protein Degradation Dissects Nanog Function During ESC Differentiation

**DOI:** 10.3390/ijms27219941

**Published:** 2026-11-06

**Authors:** Vladislav V. Vasilin, Anna S. Zinovyeva, Mikhail N. Gordeev, Ekaterina E. Petrenko, Anna S. Tsimokha, Nikolay D. Aksenov, Alexey N. Tomilin, Evgeny I. Bakhmet

**Affiliations:** Institute of Cytology, Russian Academy of Sciences, St. Petersburg 194064, Russia; v.vasilin@incras.ru (V.V.V.); a.zinoveva@incras.ru (A.S.Z.); sollussekundas@gmail.com (M.N.G.); petrenko.kate@icloud.com (E.E.P.); atsimokha@incras.ru (A.S.T.); aksenovn@gmail.com (N.D.A.); a.tomilin@incras.ru (A.N.T.)

**Keywords:** dTAG, protein degradation, ESCs, pluripotency, embryoid bodies, Nanog, FoxA2, differentiation, specification, early development

## Abstract

Protein degradation systems, such as auxin-inducible degron (AID) and dTAG-mediated degradation, have become essential tools in molecular biology. Their primary advantage lies in the rapid and efficient elimination of proteins, which facilitates the study of short-term molecular perturbations. However, these techniques remain underutilized for investigating protein function during the dynamic process of embryonic stem cell (ESC) differentiation. Here, we employed these methods to unveil the role of Nanog during definitive endoderm specification. We demonstrate that dTAG-inducible degradation is significantly more efficient than the AID system for Nanog elimination. Using this approach, we show that Nanog is a context-dependent regulator; while it maintains pluripotency in undifferentiated ESCs, it promotes definitive endoderm maturation during spontaneous differentiation. Our results highlight the advantages of targeted protein degradation for studying developmental transitions and provide new insights into the multifaceted functions of Nanog.

## 1. Introduction

The most common approach to assess the function of a specific protein is its elimination via gene knockout or knockdown. While these methods are widely used, they have several limitations, including the relatively long time required for protein depletion, ranging from hours to days. In contrast, inducible protein degradation systems, which enable rapid protein elimination, are becoming increasingly popular [1]. These approaches involve the incorporation of short peptide sequences (degrons) at the N- or C-terminus of the target protein, thereby enabling its inducible ubiquitination and subsequent proteasomal degradation. The most widely used systems include auxin-inducible degradation (AID) [2] and dTAG-mediated degradation [3]. The AID system additionally requires genomic integration of an auxin receptor, such as the F-box proteins TIR1 or AFB, and is activated by the plant hormone auxin [4]. In contrast, dTAG-mediated degradation is induced by small molecules such as dTAG-v1 or dTAG-v13, which target proteins fused to the FKBP^F36V^ degron. Notably, this system does not require additional genetic modification beyond degron tagging, as it utilizes endogenous E3 ubiquitin ligases, including VHL and CRBN [3,5]. Furthermore, auxin is typically dissolved in NaOH, whereas dTAG compounds require DMSO as a solvent, and the dTAG system has been successfully applied in vivo during embryogenesis [6].

Protein degradation approaches have been employed in several studies investigating the rapid elimination of pluripotency factors in embryonic stem cells (ESCs). These studies primarily focused on chromatin and epigenetic changes following short-term depletion of Oct4, Sox2, and Nanog. For example, Oct4 depletion leads to a decrease in H3K27ac at enhancers associated with pluripotency genes [7]. Rapid depletion of Oct4 and Sox2, but not Nanog, results in impaired cohesin binding at target sites and subsequent heterochromatin formation [8,9].

Numerous studies suggest that Oct4, Sox2, and Nanog function not only in maintaining pluripotency but also in directing lineage specification. We and others have shown that Oct4 promotes mesodermal specification [10,11], whereas Sox2 biases ESCs toward a neuroectodermal fate [12,13]; moreover, their expression levels can predict these differentiation outcomes [14,15]. Oct4 is also required for proper craniofacial development, anterior–posterior axis formation, and primordial germ cell viability during embryogenesis [11,16,17]. Nanog function has been linked to definitive endoderm specification through positive regulation of *Eomes* [18] and repression of *Pou3f1* [19]. More recently, Nanog has been shown to facilitate exit from pluripotency via repression of Sox2 during gastrulation [20].

In our view, protein degradation approaches remain underutilized for studying pluripotency factor function during ESC differentiation. These methods allow precise temporal control, enabling protein depletion at the onset of differentiation while maintaining normal expression in undifferentiated ESCs. Here, we demonstrate that dTAG-mediated degradation provides more efficient and stable depletion of Nanog compared to the AID system. Furthermore, we show that definitive endoderm formation depends on Nanog reactivation at stages corresponding to gastrulation, consistent with previous reports on its role during ESC differentiation.

## 2. Results

### 2.1. Optimization of a Strategy for Rapid Nanog Degradation

To establish an ESC line with inducible Nanog depletion, we first implemented the auxin-inducible degradation (AID) system using the miniIAA7 degron, which exhibits low basal activity [4]. Using CRISPR/Cas9, the *miniIAA7* sequence was integrated at the 3′ end of the endogenous *Nanog* alleles, and the auxin receptor gene *AtAFB2* was inserted into the actively transcribed *Rosa26* locus. Immunocytochemistry and Western blot analyses revealed a reduction in Nanog levels, with maximal depletion observed after 2 h of auxin treatment (Supplementary Figure S1a,b). However, degradation was incomplete, and Nanog levels partially recovered over time, reaching approximately 37% of the initial level after 24 h (Supplementary Figure S1b). Neither subcloning nor de novo generation of additional cell lines improved degradation efficiency.

**Figure 1:**
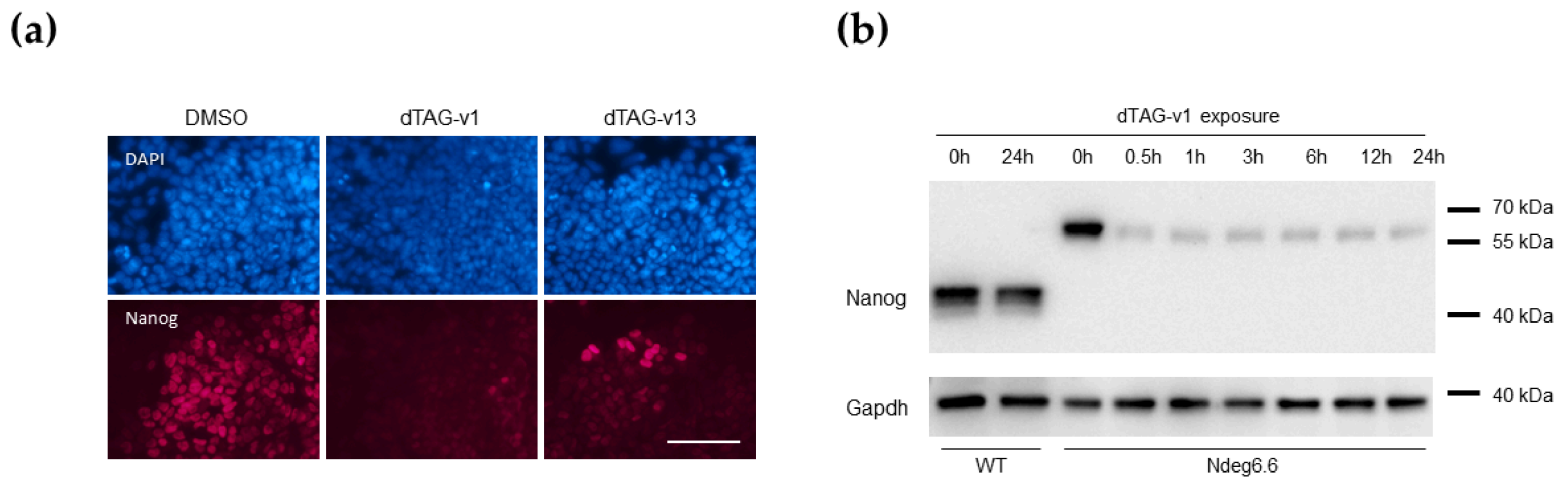
dTAG-inducible Nanog degradation in ESCs. (**a**) Immunocytochemical analysis of Nanog expression in the Ndeg6 ESC line following 2 h of treatment with either DMSO (control) or dTAG compounds. Scale bar, 100 μm. (**b**) Western blot analysis showing efficient Nanog degradation at different time points following dTAG-v1 treatment. The expected size of tagged Nanog is about 56 kDa.

We next applied the dTAG-inducible protein degradation system, which has previously been used for Nanog targeting [8]. Following integration of the *FKBP*^F36V^ degron into the *Nanog* locus, we tested the commonly used compounds dTAG-v1 and dTAG-v13. Immunocytochemistry demonstrated a strong reduction in Nanog signal after 2 h of treatment in clone 6 (Ndeg6), with dTAG-v1 showing higher efficiency and therefore used in subsequent experiments (Figure 1a). This clone was further subcloned (Ndeg6.6), and degradation kinetics were assessed by Western blot (Figure 1b). In contrast to the AID system, Nanog levels were reduced to approximately 14% of baseline as early as 30 min after dTAG treatment and further to 10%. Notably, this level of depletion was maintained for at least 24 h (Figure 1b). Although complete elimination of Nanog was not achieved, this is consistent with previously reported efficiencies of the dTAG system for this protein [8].

To investigate Nanog function during ESC differentiation toward definitive endoderm, we generated a reporter cell line (F39T4_Ndeg18) carrying a T2A-EGFP cassette in the *FoxA2* locus, a T2A-tagBFP cassette in the *T* (*Brachyury*) locus, and the FKBP^F36V^ degron fused to Nanog. Nanog degradation efficiency in this line was comparable to that observed in the Ndeg6.6 line (Supplementary Figure S2a,b).

### 2.2. Embryoid Bodies as a Model of Gastrulation

Prior to investigating Nanog function in definitive endoderm specification, we first characterized the expression dynamics of pluripotency and lineage specification markers during embryoid body (EB) differentiation, a widely used in vitro model of early embryonic development.

**Figure 2:**
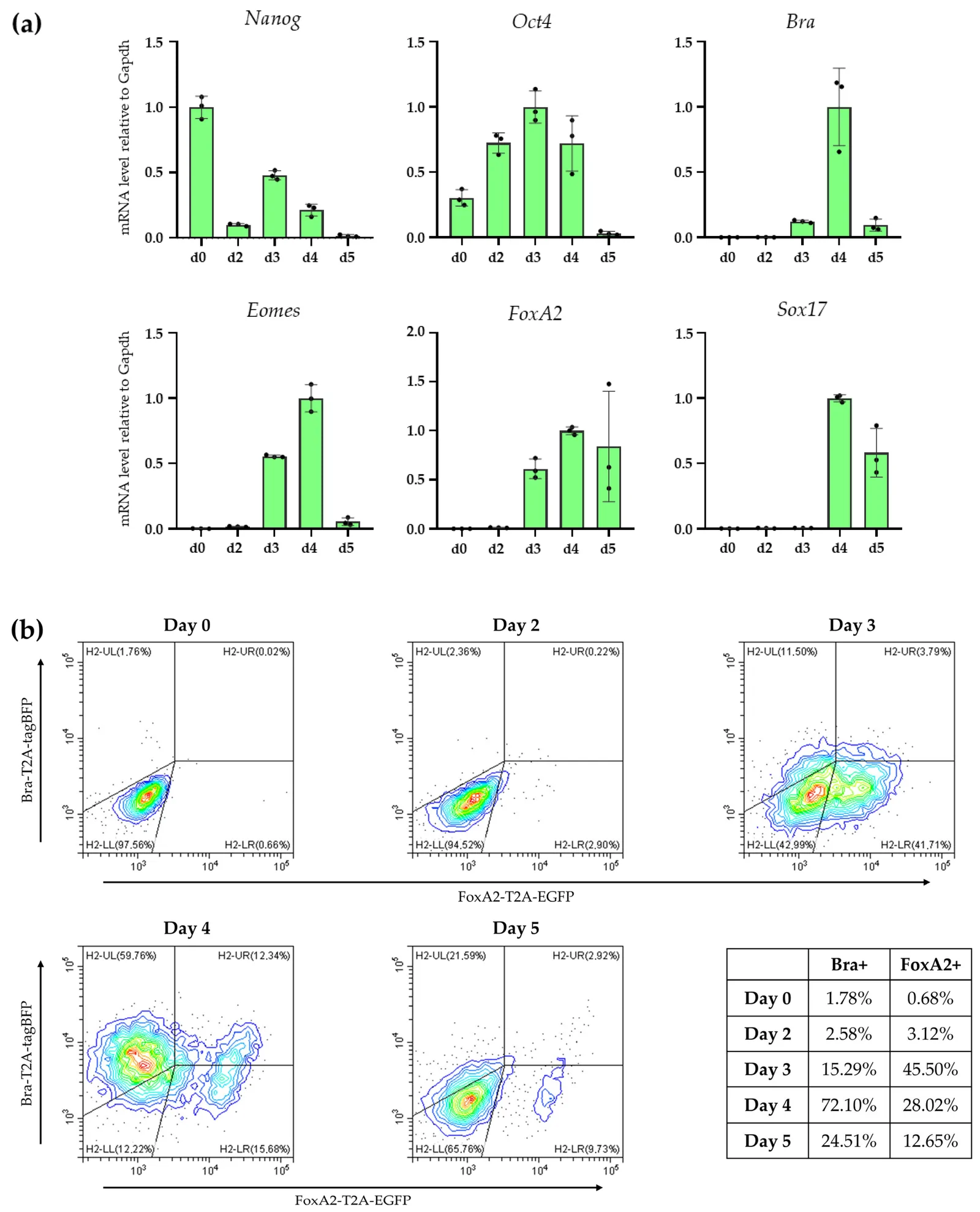
Dynamics of pluripotency, endodermal, and primitive streak marker expression during spontaneous EB differentiation. (**a**) Relative mRNA expression levels of *Nanog*, *Oct4*, *Sox17*, *FoxA2*, *Eomes*, and *Brachyury* on Day 0 (ESCs) and Days 2–5 of differentiation. n = 3 technical replicates. Error bars indicate the standard deviation (SD). (**b**) Left panel: flow cytometry analysis of the F39T4_Ndeg18 cell line on Day 0 and Days 2–5 of differentiation. Right panel: table showing the percentages of Bra^+^ (BFP) and FoxA2^+^ (EGFP) cells. Contour plots reflect density of cells within certain area from blue (low) to red (high).

Consistent with its in vivo expression pattern [20,21,22] and our previous in vitro observations [23], *Nanog* expression was downregulated by Day 2, corresponding to the formative pluripotent state, and subsequently re-expressed on Day 3, coinciding with the onset of gastrulation-like events (Figure 2a). In parallel, *Oct4* levels increased up to Day 3, in line with its role in mesodermal specification [10]. As expected, primitive streak markers *Eomes* and *Brachyury* were induced on Day 3 and reached peak expression on Day 4, followed by upregulation of the definitive endoderm markers *Sox17* and *FoxA2*.

Consistent with RT-PCR results, flow cytometry analysis revealed peak expression of *Brachyury* and *FoxA2* on Days 3 and 4 of differentiation (Figure 2b). Notably, a distinct *FoxA2*^+^ cell population emerged on Day 4, suggesting further progression toward definitive endoderm specification (Figure 2b).

Together, these data indicate a coordinated and dynamic interplay between pluripotency, primitive streak, and endodermal markers during EB differentiation.

### 2.3. Nanog Degradation Impairs Definitive Endoderm Differentiation

**Figure 3:**
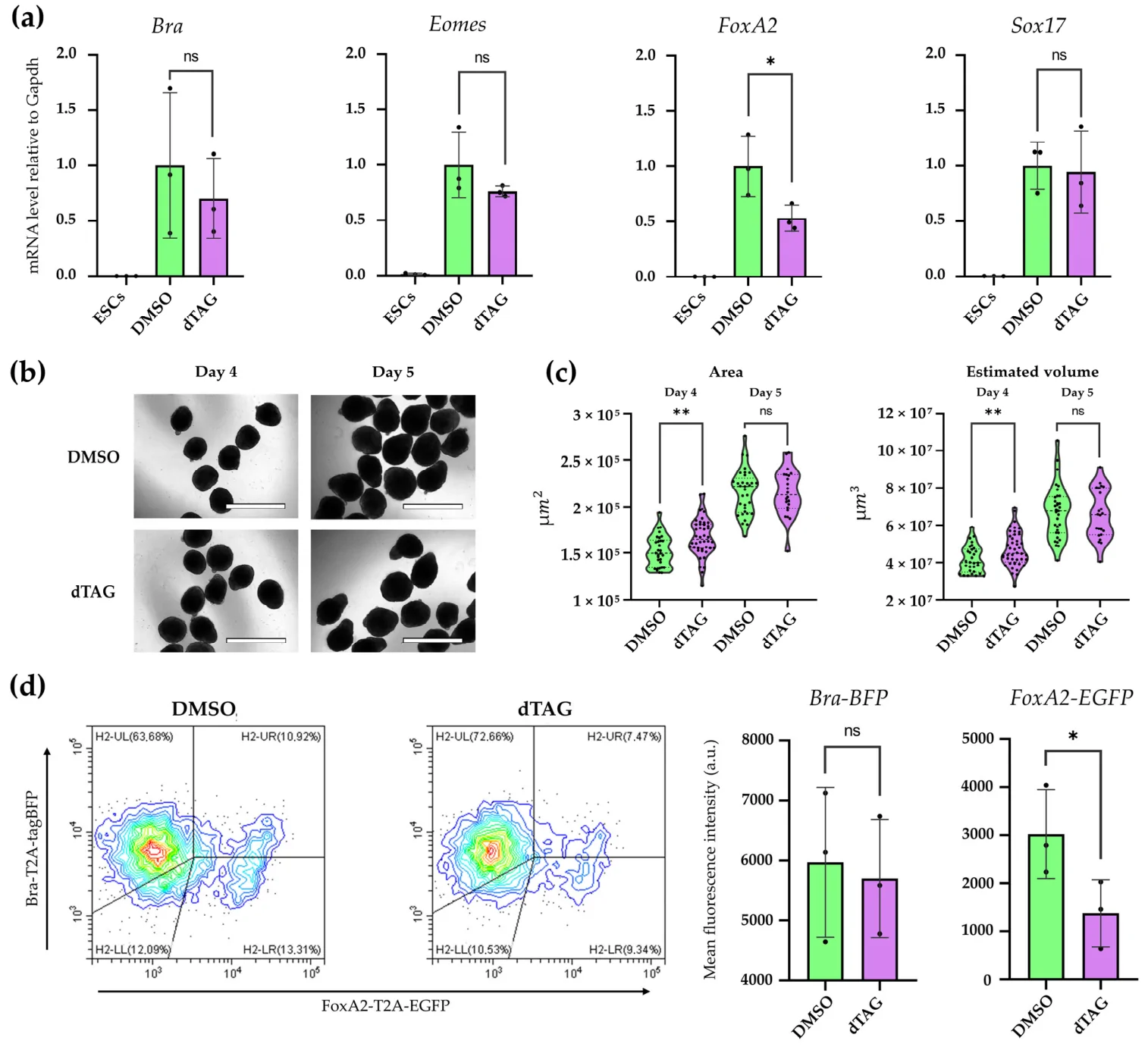
Effect of Nanog degradation on definitive endoderm and primitive streak marker expression. (**a**) qPCR analysis of key mesendodermal markers at Day 4 of differentiation following treatment with DMSO (control) or dTAG from Day 2. Gene expression levels were normalized to *Gapdh* and expressed relative to the DMSO control. N = 3 biological replicates. Error bars indicate standard deviation (SD). (**b**) Bright-field images of embryoid bodies (EBs) on Days 4–5 of differentiation. Scale bar, 1000 μm. (**c**) Quantification of EB area and volume at Days 4–5 of differentiation. Volume was calculated from the measured two-dimensional area by assuming a spherical shape. A significant difference was observed on Day 4 only. At least 22 EBs were analyzed. (**d**) Representative flow cytometry plots of cells from EBs showing a decrease in the FoxA2^+^ population in the dTAG-treated condition compared to control. Contour plots reflect density of cells within certain area from blue (low) to red (high). Bar graphs show background-corrected mean fluorescence intensity (MFI) for each reporter. Background correction was performed by subtracting the corresponding Day 0 (ESC) MFI from the Day 4 MFI for each independent experiment. Data represent three independent biological replicates (N = 3). Error bars indicate SD. ns, not significant; * *p* < 0.05; ** *p* < 0.01.

To investigate the role of Nanog in definitive endoderm specification, we induced its degradation from Day 2 of EB formation and analyzed the expression of lineage specification markers on Day 4 (Figure 3a). qPCR analysis revealed that *Bra*, *Eomes*, and *Sox17* expression levels were not significantly affected by Nanog degradation, whereas *FoxA2* mRNA levels were significantly reduced.

We next examined EB morphology on Days 4 and 5 (Figure 3b). Unexpectedly, EBs subjected to Nanog degradation were larger on Day 4; however, this difference was no longer observed by Day 5 (Figure 3c).

Flow cytometry analysis on Day 4 revealed a significant reduction in FoxA2–EGFP reporter fluorescence intensity following dTAG treatment, whereas Bra–BFP fluorescence intensity was not significantly affected (Figure 3d). Representative flow cytometry plots also showed a reduced proportion of FoxA2–EGFP-positive cells in dTAG-treated EBs, consistent with the qPCR results.

Together, these findings suggest that Nanog contributes to *FoxA2* expression during EB differentiation, while the examined primitive streak-associated markers are not significantly affected under these conditions.

**Figure 4:**
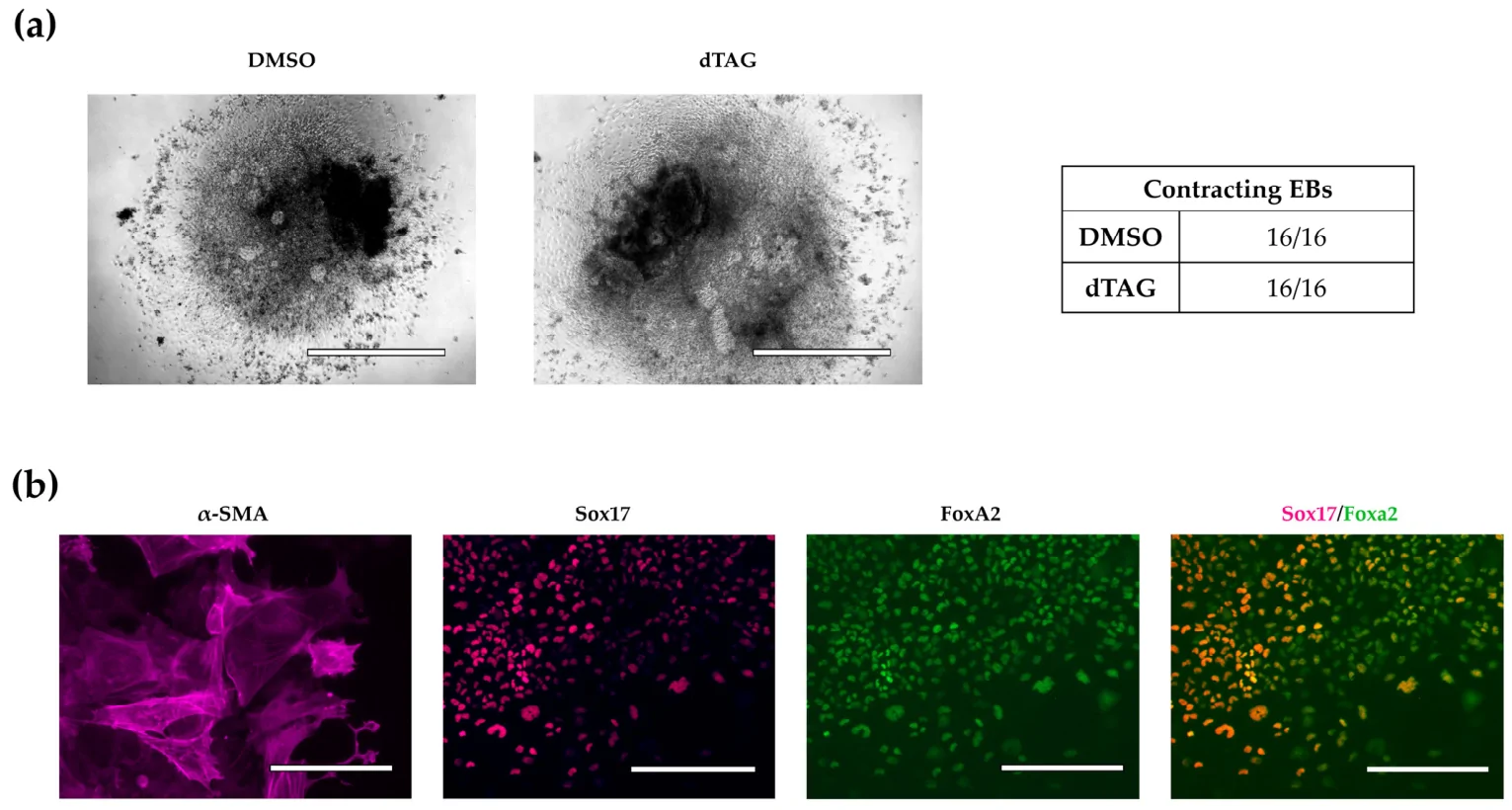
Nanog degradation does not prevent differentiation into multiple lineages in embryoid bodies. (**a**) Bright-field images of EB outgrowths derived from control and dTAG-treated conditions (**left**). Scale bar, 1000 μm. Quantification of contracting outgrowths relative to the total number of embryoid bodies under each condition (**right**). (**b**) Immunocytochemical analysis of EB outgrowths showing α-SMA–positive cells (scale bar, 100 μm) and Sox17/FoxA2-double positive cells (scale bar, 400 μm). The right panel shows a merged image of Sox17 and FoxA2.

Next, we performed morphological and immunocytochemical analyses of EB outgrowths following dTAG treatment. EB morphology, as well as the proportion of contracting structures, was not affected by Nanog degradation, indicating successful mesodermal differentiation (Figure 4a).

Immunocytochemistry analysis demonstrated the presence of α-SMA-positive cells, further supporting normal mesodermal specification (Figure 4b). Similarly, FoxA2/Sox17 co-expression, consistent with the qPCR results showing reduced but still clearly detectable expression levels, indicated activation of the definitive endoderm differentiation program in EBs following Nanog degradation.

Together, these results suggest that Nanog promotes definitive endoderm development by positively regulating the expression of key markers, although it is not a direct inducer of this lineage.

## 3. Discussion

In this study, we demonstrate that dTAG-inducible degradation can be successfully used to investigate the functions of pluripotency factors during ESC differentiation. In contrast to conditional knockout approaches—which we recently used to study Oct4 function during mesodermal specification [10]—this technique enables rapid depletion of a protein of interest within short periods of up to 30 min. This feature facilitates the investigation of a protein function during defined temporal windows and may therefore help distinguish between early and late roles of regulatory proteins during dynamic differentiation processes. Interestingly, under the experimental conditions used here, dTAG-inducible degradation was more efficient than the AID for Nanog depletion. Importantly, dTAG-mediated degradation showed rapid and prolonged elimination that remained consistent from 30 min to 24 h. However, Nanog degradation was incomplete, which limits further interpretation of the results. Consequently, we cannot exclude the possibility that the residual approximately 10% of Nanog contributes to the maintenance of regulatory networks associated with primitive streak or endodermal markers. The present data do not allow us to determine the minimal Nanog level required to maintain such regulatory activity. The relatively rapid turnover of Nanog has been reported previously [8] and may be related to its relatively short half-life of approximately 2 h [24].

New evidence continues to emerge regarding the functions of pluripotency factors during ESC differentiation. Although many studies have investigated the depletion or overexpression of Oct4, Sox2, and Nanog in ESCs [12,13,25,26,27], relatively few have focused on their functions directly during differentiation [11,15,28,29]. Interestingly, in contrast to the previously proposed positive regulation of the primitive streak marker *Eomes* by Nanog [18], we did not detect a statistically significant change in *Eomes* expression following Nanog degradation in Day 4 EBs. Instead, Nanog degradation significantly reduced *FoxA2* mRNA expression and FoxA2–EGFP reporter fluorescence, suggesting a role for Nanog in regulating *FoxA2* expression during EB differentiation. This apparent discrepancy with previous findings may reflect differences in experimental conditions or incomplete Nanog degradation, potentially due to limited penetration of the dTAG compound into EBs.

Overall, our results suggest that Nanog may contribute to definitive endoderm maturation, as indicated by the trends observed in RT-PCR and reporter assays. These findings are consistent with the idea that Nanog—often considered a “guardian” of the undifferentiated state [13,25] —may perform context-dependent functions during differentiation. Once gastrulation-associated differentiation is initiated, Nanog may participate in regulatory processes that promote exit from pluripotency and subsequent lineage specification [20]. Further studies should clarify the molecular mechanisms underlying this context-dependent change in Nanog activity and determine whether it is associated with Nanog modifications that mediate interactions with other proteins [30], or with Nanog homodimerization [31].

We also observed a transient increase in embryoid body size on Day 4, but not on Day 5, following Nanog degradation. This effect may reflect transient changes in cell proliferation or other cellular processes affecting embryoid body growth and organization. It may also be associated with changes in lineage composition, although the observed trends in primitive streak-associated markers were modest. Because these processes were not directly assessed in the present study, the mechanisms underlying this transient morphological change remain unclear.

Overall, this study primarily highlights the applicability of dTAG-inducible degradation for investigating protein functions during differentiation while preserving normal expression of the target protein in ESCs before induction of degradation. Additional work will be required to clarify the mechanisms by which Nanog may influence definitive endoderm maturation. In particular, it remains to be determined whether this effect involves direct regulation of endoderm-associated genes or instead results indirectly from modulation of the pluripotency regulatory network or alternative lineage programs during exit from pluripotency. These questions can be addressed using chromatin-binding profiling and RNA-seq approaches in future studies. Further investigation using a defined direct differentiation system will also be required to determine whether the effects observed here extend beyond the embryoid body model. Finally, the established cell lines may provide a useful platform for investigating Nanog function during embryogenesis, as dTAG-inducible degradation is well suited for temporally controlled protein depletion in such experimental settings [32].

## 4. Materials and Methods

### 4.1. Plasmid Construction

Plasmids for the Auxin-Inducible Degron (AID) system were constructed using Golden Gate assembly. The Benchling platform (Benchling, Inc., San Francisco, CA, USA; benchling.com) was utilized for plasmid design. Left and right homology arms (705 bp and 648 bp, respectively), corresponding to the regions adjacent to the *Nanog* stop codon, were amplified from mouse genomic DNA. The sequences of both homology arms were modified to eliminate the native stop codon and the PAM site. The miniIAA7 degron (with a GS-linker and IRES-BSD) was amplified from Addgene (Watertown, MA, USA) plasmid #129719, and the 3xFlag tag was amplified from Addgene #42230. The plasmid backbone was amplified from Addgene #140540.

All PCR reactions were performed using Phusion DNA polymerase (Thermo Fisher Scientific, Waltham, MA, USA). Subsequently, 3′-A overhangs were added to the miniIAA7 degron, IRES-BSD, 3xFlag, and *Nanog* homology arms; these fragments were then ligated into the pKAN-T vector (Evrogen, Moscow, Russia). Correct plasmids were combined with a purified backbone in a Golden Gate reaction using 15 U of BsmBI (New England Biolabs, Ipswich, MA, USA) and 500 U of T4 ligase (SibEnzyme, Novosibirsk, Russia). The reaction was performed in a thermocycler for 30 cycles (5 min at 37 °C, followed by 5 min at 16 °C). The CRISPR guide RNA (sgRNA) targeting the *Nanog* stop codon was designed using Benchling, with sequences selected based on on-target and off-target scores [33]. The chosen guide was purchased from Evrogen (Russia), annealed, and ligated into the LentiCRISPRv2 vector (#52961), resulting in the establishment of the LCV2_Nanog-3′ plasmid.

A two-stage cloning strategy was used to generate the donor construct for AtAFB2 insertion into the *Rosa26* locus. First, the Rosa26-TRE-Frt-ERT2CreERT2-STOP-Frt-tdTomato plasmid was digested with MluI (New England Biolabs, USA) and BbvCI (New England Biolabs, USA) to remove the ERT2CreERT2-STOP-Frt-tdTomato sequence. A PCR fragment containing a new MfeI restriction site was then ligated in its place. Subsequently, the AtAFB2 sequence was amplified from Addgene plasmid #129717, with a weak nuclear localization signal (NLS) fused to the C-terminus. The fragment was then ligated into the modified vector via the MfeI and MluI restriction sites. The resulting Rosa26-AtAFB2-weakNLS plasmid was used in all subsequent experiments. To facilitate knock-in at the *Rosa26* locus, a pX330-based plasmid was used.

Two plasmids were generated to introduce the FKBP12^F36V^ degron at the C-terminus of Nanog. The Cas9/sgRNA plasmid was constructed by cloning Nanog-targeting sgRNA (Table 1) into the lentiCRISPRv2 vector (Addgene #52961) digested with Esp3I (NEB). The HDR (Homology-directed repair) donor plasmid, Nanog_FKBP_2HA—containing the FKBP12^F36V^ tag, a 2xHA tag, a blasticidin S resistance cassette, and an S80 poly(A) signal flanked by a 688 bp Nanog 5′ left homology arm and a 621 bp Nanog 3′ right arm—was assembled. An intermediate vector containing FKBP12^F36V^-GSG-mCherry and the blasticidin S resistance cassette flanked by Nanog homology arms was first constructed. The FKBP12^F36V^ sequence was assembled from four synthesized fragments cloned into pJET1.2/blunt and combined by Golden Gate cloning. The blasticidin S resistance cassette and Nanog homology arms were pre-cloned into the pKAN-T vector (Evrogen), and all elements were assembled by Golden Gate cloning. The final HDR donor was generated by around-the-horn PCR to replace mCherry with 2xHA using primers containing EcoRI sites (Table 1). The resulting amplicon was ligated and used for transfection.

To generate the F39T4 reporter ESC line, previously established Foxa2-T2A-EGFP [23] and Bra-T2A-tagBFP [10] constructs, along with their corresponding gRNA sequences, were utilized.

### 4.2. ESC Lines

E14 Tg2a murine ESCs (Bay Genomics, Berkeley, CA, USA) were used as the wild-type (WT) ESC line. To generate the F39T4 double-reporter ESC line, Foxa2-T2A-EGFP and Bra-T2A-tagBFP donor vectors, along with LentiCRISPRv2 plasmids harboring the corresponding sgRNAs (500 ng at a 1:1 molar ratio), were transfected using FuGENE HD transfection reagent (Promega, Madison, WI, USA) in OptiMEM medium. The following day, cells were transferred onto a 10-cm gelatinized dish. One day later, 2 μg/mL puromycin (Merck, Darmstadt, Germany) was added to the culture medium for two days. The cells were then cultured for an additional 10 days without selective antibiotics. Single clones were subsequently picked, expanded, and screened for transgene insertion via PCR using previously reported primers [10,23] and the LR HS-PCR kit (Biolabmix, Novosibirsk, Russia).

To generate the Ndeg6.6 and F39T4_Ndeg18 ESC lines with inducible Nanog degradation, WT or F39T4 ESCs were transfected with the Nanog_FKBP_2HA donor and LCV2_Nanog-3′ plasmids. Cell clones were picked as described above. Nanog degradation efficiency was verified using 0.5 µM dTAG-v1 or dTAG-v13 (Tocris Bioscience, Bristol, UK), followed by immunocytochemical analysis.

### 4.3. Cell Culture

Unless otherwise specified, all reagents for mouse ESC culture were purchased from Gibco (Thermo Fisher Scientific, Waltham, MA, USA). ESCs were routinely cultured on adhesive plastic dishes (Eppendorf, Hamburg, Germany or TPP, Trasadingen, Switzerland) pre-coated with 0.1% gelatin (Merck) under standard conditions (5% CO_2_, 37 °C). The mES medium consisted of Knockout DMEM supplemented with 15% fetal bovine serum (HyClone, Cytiva, Logan, UT, USA), 1× penicillin/streptomycin, 2 mM L-glutamine, 1× non-essential amino acids, 50 μM β-mercaptoethanol, and 500 U/mL bacterially expressed human LIF produced in-house. Passaging was performed using 0.05% Trypsin-EDTA.

Prior to experiments, ESC lines were pre-cultured for at least 6 days in medium containing LIF and 1 μM PD0325901 (Axon, Scottsdale, AZ, USA) to stabilize the pluripotent state and eliminate spontaneous differentiation. Nanog degradation was initiated by the addition of dTAG-v1 or dTAG-v13 (Tocris) to a final concentration of 0.5 µM.

### 4.4. EB Formation and Differentiation

Single-cell suspensions of ESCs were prepared by dissociation with 0.05% Trypsin-EDTA and seeded at 600 cells per 35 μL hanging drop on the lid of a 10-cm Petri dish to initiate embryoid body (EB) formation (Day 0). Sterile dH2O was added to the Petri dish to maintain adequate humidity during differentiation. The culture medium for EB generation was identical to that used for ESC maintenance, except that LIF and PD0325901 were omitted. After 48 h (Day 2), DMSO (control) or dTAG-v1 was added to the EBs to induce Nanog degradation. Where indicated, at Day 5, EBs were plated onto 48-well gelatin-coated plates in DMEM/F12 GlutaMAX supplemented with 10% FBS and penicillin/streptomycin. EBs were cultured for an additional 3 days to monitor spontaneous contractile activity. Beating EBs were recorded via screen capture using an EVOS FL Auto microscope (Thermo Fisher Scientific) and subsequently fixed for immunocytochemical analysis.

### 4.5. Flow Cytometry

Flow cytometric analyses were performed to assess the dynamics of reporter gene expression in F39T4 ESCs during the spontaneous differentiation of ESCs into Ebs. Analysis was conducted using a CytoFLEX benchtop flow cytometer (Beckman Coulter, Brea, CA, USA) equipped with 405-nm and 488-nm lasers. The 405-nm laser was used for tagBFP detection, and the 488-nm laser was used for EGFP detection (FITC channel). Fluorescence intensities were measured at multiple time points (Day 0 to Day 5) during differentiation. Debris and doublets were excluded via forward/side scatter gating. All data were processed using CytExpert software (version 2.4, Beckman Coulter).

### 4.6. Immunocytochemistry and Imaging

Cells were fixed with 4% paraformaldehyde for 10 min directly on culture plates, washed with PBS containing 0.1% Tween-20 (PBST), permeabilized with 0.1% Triton X-100 for 15 min, and blocked with 3% BSA-PBST for 30 min. Incubation with primary antibodies (diluted in 3% BSA-PBST) was performed for 2 h at room temperature or overnight at 4 °C. Cells were washed 5–6 times with PBST and incubated with secondary fluorescent antibodies (diluted in 3% BSA-PBST) for 1 h at room temperature. Subsequently, cells were washed 3 times with PBST, incubated with DAPI for 2 min, washed again, and stored in PBS with sodium azide.

All primary antibodies used in this study are listed in Table 2. Secondary fluorescent antibodies were purchased from Jackson ImmunoResearch (West Grove, PA, USA). Fluorescence microscopy was performed using an EVOS FL Auto (Thermo Fisher Scientific) microscope equipped with DAPI, GFP, RFP, and Cy5 filter cubes.

### 4.7. Gene Expression Analysis by Real-Time Quantitative PCR

Total RNA was isolated from cells using the guanidinium thiocyanate–phenol–chloroform extraction method with the RNA-Extract reagent (Evrogen, Russia) according to the manufacturer’s instructions. Complementary DNA (cDNA) was synthesized from 2.5 µg of total RNA using random hexamer primers and MMLV reverse transcriptase (Evrogen, Russia).

Real-time PCR was performed with 5× qPCRmix-HS SYBR (Evrogen, Russia) on an ABI 7500 Real-Time PCR System (Thermo Fisher Scientific, USA). Gene expression levels were normalized to *Gapdh* expression. Primer sequences are listed in Table 3.

### 4.8. Statistical Analysis

Statistical analyses were performed using the GraphPad Prism v8.4.2 package (GraphPad Software, Boston, MA, USA). Differences between groups were evaluated using an unpaired *t*-test with Welch’s correction for embryoid body morphology and a two-tailed paired *t*-test for RT-qPCR and flow cytometry data. Error bars indicate the standard deviation (SD); * *p* < 0.05, ** *p* < 0.01.

Quantitative analysis of EB morphology was performed using Fiji (version 1.54, ImageJ, NIH, Bethesda, MD, USA). The area and volume of individual EBs were measured manually from bright-field micrographs acquired on Day 4 and Day 5 of differentiation. Each EB was outlined using the freehand selection tool, and its area was recorded automatically. EB volume was estimated assuming spherical geometry based on the measured two-dimensional area. For each experimental condition, at least 20 EBs were analyzed per biological replicate.

## Figures and Tables

**Table 1:** Oligonucleotides used for cloning.

Gene	Primer Sequence (Forward)
*Nanog 3′ gRNA*	TATGAGACTTACGCAACATC
*FKPB.V_part_I*	attcgcggtctccttcacgaacgtctccataggaagcggaggagtgcaggtggaaaccatctccccaggcgacgggcgcaccttccccaagcgcggccagacctgcgtggagaccgcgaat
*FKPB.V_part_II*	attcgcggtctcggcgtggtgcactacaccgggatgcttgaagatggaaagaaagttgattcctcccgggacagaaacaagccctttaagtttatgctaggcaagcaggacgagaccgcgaat
*FKPB.V_part_III*	attcgcggtctcgaggaggtgatccgaggctgggaagaaggggttgcccagatgagtgtgggtcagagagccaaactgactatatctccagattatgcctatggtgccacgagaccgcgaat
*FKPB.V_part_IV*	attcgcggtctccgccactgggcacccaggcatcatcccaccacatgccactctcgtcttcgatgtggagcttctaaaactggaaggaagcggaatggagacgttcgtgtgggagaccgcgaat
*R2_2HA_stop_EcoRI*	tagcgaattcttatgcatagtccgggacatcatacggatagccggcgtagtcgggcacgtcgtaggggtagccgccttccagttttagaagctccaca
*F_2HA_EcoRI*	tgcagaattcgaccgctctagcccaattccgc

**Table 2:** List of primary antibodies used in this study.

Target	Cat. No.	Manufacturer
Nanog	A300-397	Bethyl, Montgomery, TX, USA
α-SMA	A2547	Sigma-Aldrich, St. Louis, MO, USA
Sox17	AF1924	R&D, Fort Worth, TX, USA
FoxA2	sc-374375	Santa Cruz Biotechnology, Dallas, TX, USA

**Table 3:** Primer Sequences.

Gene	Primer Sequence (Forward)	Primer Sequence (Reverse)
*Oct4A*	GGCTTCAGACTTCGCCTTCT	TGGAAGCTTAGCCAGGTTCG
*Brachyury*	GGCTGGGAGCTCAGTTCTTT	AGCAGCCCCTTCATACATCG
*Eomes*	AAGCTCAAGAAAGGAAACATGC	ACCGGCACCAAACTGAGA
*FoxA2*	CATGAACTCGATGAGCCCCA	TGTAGCTGCGTCGGTATGTC
*Sox17*	CACAACGCAGAGCTAAGCAA	CGCTTCTCTGCCAAGGTC
*Nanog*	GCTCCATAACTTCGGGGAGG	GTGCTAAAATGCGCATGGCT
*Gapdh*	AGGTCGGTGTGAACGGATTTG	TGTAGACCATGTAGTTGAGGTCA

## Data Availability

No new data were created or analyzed in this study.

## Supplementary Materials

The following supporting information can be downloaded at: https://www.mdpi.com/article/10.3390/ijms27219941/s1.

## References

[1] Tsai J.M., Nowak R.P., Ebert B.L., Fischer E.S. (2024). Targeted protein degradation: From mechanisms to clinic. Nat. Rev. Mol. Cell Biol..

[2] Nishimura K., Fukagawa T., Takisawa H., Kakimoto T., Kanemaki M. (2009). An auxin-based degron system for the rapid depletion of proteins in nonplant cells. Nat. Methods.

[3] Nabet B., Roberts J.M., Buckley D.L., Paulk J., Dastjerdi S., Yang A., Leggett A.L., Erb M.A., Lawlor M.A., Souza A. (2018). The dTAG system for immediate and target-specific protein degradation. Nat. Chem. Biol..

[4] Li S., Prasanna X., Salo V.T., Vattulainen I., Ikonen E. (2019). An efficient auxin-inducible degron system with low basal degradation in human cells. Nat. Methods.

[5] Nabet B., Ferguson F.M., Seong B.K.A., Kuljanin M., Leggett A.L., Mohardt M.L., Robichaud A., Conway A.S., Buckley D.L., Mancias J.D. (2020). Rapid and direct control of target protein levels with VHL-recruiting dTAG molecules. Nat. Commun..

[6] Abuhashem A., Lee A.S., Joyner A.L., Hadjantonakis A.K. (2022). Rapid and efficient degradation of endogenous proteins in vivo identifies stage-specific roles of RNA Pol II pausing in mammalian development. Dev. Cell.

[7] Bates L.E., Alves M.R.P., Silva J.C.R. (2021). Auxin-degron system identifies immediate mechanisms of OCT4. Stem Cell Rep..

[8] Liu N.Q., Maresca M., van den Brand T., Braccioli L., Schijns M., Teunissen H., Bruneau B.G., Nora E.P., de Wit E. (2021). WAPL maintains a cohesin loading cycle to preserve cell-type-specific distal gene regulation. Nat. Genet..

[9] Maresca M., van den Brand T., Li H., Teunissen H., Davies J., de Wit E. (2023). Pioneer activity distinguishes activating from non-activating SOX2 binding sites. EMBO J..

[10] Lukacheva A.V., Zinovyeva A.S., Kuzmin A.A., Gordeev M.N., Vasilin V.V., Kriger D.V., Aksenov N.D., Tomilin A.N., Bakhmet E.I. (2025). Oct4 Contributes to Mesodermal Differentiation by Sustaining the Proliferative Capacity of Early Mesodermal Progenitors. Int. J. Mol. Sci..

[11] Mulas C., Chia G., Jones K.A., Hodgson A.C., Stirparo G.G., Nichols J. (2018). Oct4 regulates the embryonic axis and coordinates exit from pluripotency and germ layer specification in the mouse embryo. Development.

[12] Kopp J.L., Ormsbee B.D., Desler M., Rizzino A. (2008). Small increases in the level of Sox2 trigger the differentiation of mouse embryonic stem cells. Stem Cells.

[13] Wang Z., Oron E., Nelson B., Razis S., Ivanova N. (2012). Distinct lineage specification roles for NANOG, OCT4, and SOX2 in human embryonic stem cells. Cell Stem Cell.

[14] Malleshaiah M., Padi M., Rue P., Quackenbush J., Martinez-Arias A., Gunawardena J. (2016). Nac1 Coordinates a Sub-network of Pluripotency Factors to Regulate Embryonic Stem Cell Differentiation. Cell Rep..

[15] Valcourt J.R., Huang R.E., Kundu S., Venkatasubramanian D., Kingston R.E., Ramanathan S. (2021). Modulating mesendoderm competence during human germ layer differentiation. Cell Rep..

[16] Kehler J., Tolkunova E., Koschorz B., Pesce M., Gentile L., Boiani M., Lomeli H., Nagy A., McLaughlin K.J., Scholer H.R. (2004). Oct4 is required for primordial germ cell survival. EMBO Rep..

[17] DeVeale B., Brokhman I., Mohseni P., Babak T., Yoon C., Lin A., Onishi K., Tomilin A., Pevny L., Zandstra P.W. (2013). Oct4 is required ~E7.5 for proliferation in the primitive streak. PLoS Genet..

[18] Teo A.K., Arnold S.J., Trotter M.W., Brown S., Ang L.T., Chng Z., Robertson E.J., Dunn N.R., Vallier L. (2011). Pluripotency factors regulate definitive endoderm specification through eomesodermin. Genes Dev..

[19] Barral A., Rollan I., Sanchez-Iranzo H., Jawaid W., Badia-Careaga C., Menchero S., Gomez M.J., Torroja C., Sanchez-Cabo F., Gottgens B. (2019). Nanog regulates Pou3f1 expression at the exit from pluripotency during gastrulation. Biol. Open.

[20] Wong F.C.K., Zhang M., Thomson E., Schumacher L.J., Tsakiridis A., Ashmore J., Li T., Blin G., Karagianni E., Mullin N.P. (2025). NANOG is repurposed after implantation to repress Sox2 and begin pluripotency extinction. EMBO J..

[21] Hart A.H., Hartley L., Ibrahim M., Robb L. (2004). Identification, cloning and expression analysis of the pluripotency promoting Nanog genes in mouse and human. Dev. Dyn..

[22] Hoffman J.A., Wu C.I., Merrill B.J. (2013). Tcf7l1 prepares epiblast cells in the gastrulating mouse embryo for lineage specification. Development.

[23] Gordeev M.N., Zinovyeva A.S., Petrenko E.E., Lomert E.V., Aksenov N.D., Tomilin A.N., Bakhmet E.I. (2024). Embryonic Stem Cell Differentiation to Definitive Endoderm As a Model of Heterogeneity Onset During Germ Layer Specification. Acta Naturae.

[24] Ramakrishna S., Suresh B., Lim K.H., Cha B.H., Lee S.H., Kim K.S., Baek K.H. (2011). PEST motif sequence regulating human NANOG for proteasomal degradation. Stem Cells Dev..

[25] Chambers I., Colby D., Robertson M., Nichols J., Lee S., Tweedie S., Smith A. (2003). Functional expression cloning of Nanog, a pluripotency sustaining factor in embryonic stem cells. Cell.

[26] Niwa H., Miyazaki J.-I., Smith A.G. (2000). Quantitative expression of Oct-3/4 defines differentiation, dedifferentiation or self-renewal of ES cells. Nat. Genet..

[27] Yu Y., Wang X., Zhang X., Zhai Y., Lu X., Ma H., Zhu K., Zhao T., Jiao J., Zhao Z.A. (2018). ERK inhibition promotes neuroectodermal precursor commitment by blocking self-renewal and primitive streak formation of the epiblast. Stem Cell Res. Ther..

[28] Thomson M., Liu S.J., Zou L.N., Smith Z., Meissner A., Ramanathan S. (2011). Pluripotency factors in embryonic stem cells regulate differentiation into germ layers. Cell.

[29] Radzisheuskaya A., Chia Gle B., dos Santos R.L., Theunissen T.W., Castro L.F., Nichols J., Silva J.C. (2013). A defined Oct4 level governs cell state transitions of pluripotency entry and differentiation into all embryonic lineages. Nat. Cell Biol..

[30] Wang D., Shi X., Xie J., Zhao L., Wu B., Dong Q., Huang Y., Suo J., Wang X., He B. (2026). WDR5 remodels NANOG condensates to drive transcriptional programs and sustain stem cell identity. Nat. Commun..

[31] Choi K.J., Quan M.D., Qi C., Lee J.H., Tsoi P.S., Zahabiyon M., Bajic A., Hu L., Prasad B.V.V., Liao S.J. (2022). NANOG prion-like assembly mediates DNA bridging to facilitate chromatin reorganization and activation of pluripotency. Nat. Cell Biol..

[32] Abuhashem A., Hadjantonakis A.K. (2022). Generation of knock-in degron tags for endogenous proteins in mice using the dTAG system. STAR Protoc..

[33] Hsu P.D., Scott D.A., Weinstein J.A., Ran F.A., Konermann S., Agarwala V., Li Y., Fine E.J., Wu X., Shalem O. (2013). DNA targeting specificity of RNA-guided Cas9 nucleases. Nat. Biotechnol..

