# Supplementary material for: dTAG-Mediated Protein Degradation Dissects Nanog Function During ESC Differentiation
Source: Int J Mol Sci. 2026 Nov 6;27(21):9941. doi: 10.3390/ijms27219941 (PMC13764645; doi:10.3390/ijms27219941)
Supplement: Supplementary file 1 [file ijms-27-09941-s001.zip › ijms-4589579-supplementary.pdf]

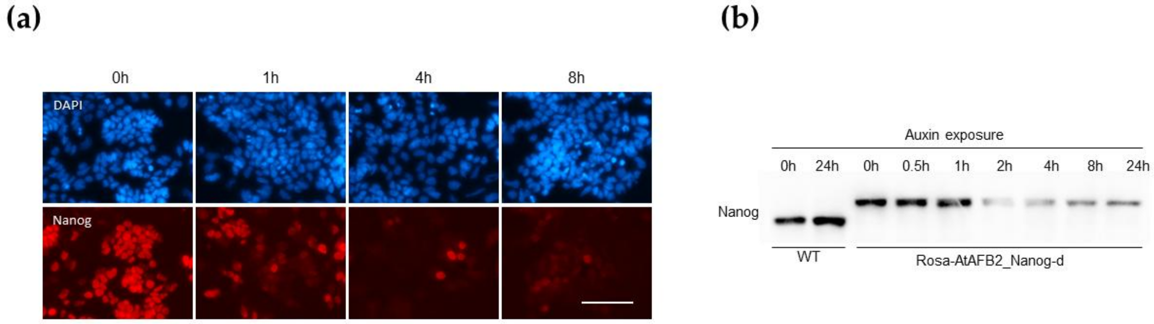

Supplementary Figure S1. Auxin-inducible (AID) Nanog degradation in ESCs.

(a) Immunocytochemical analysis of Nanog expression in the AID-Nanog ESC line following 2 hours of treatment with either DMSO (control) or dTAG compounds. Scale bar, 100 µm.
(b) Western blot analysis showing Nanog degradation at different time points following auxin treatment.

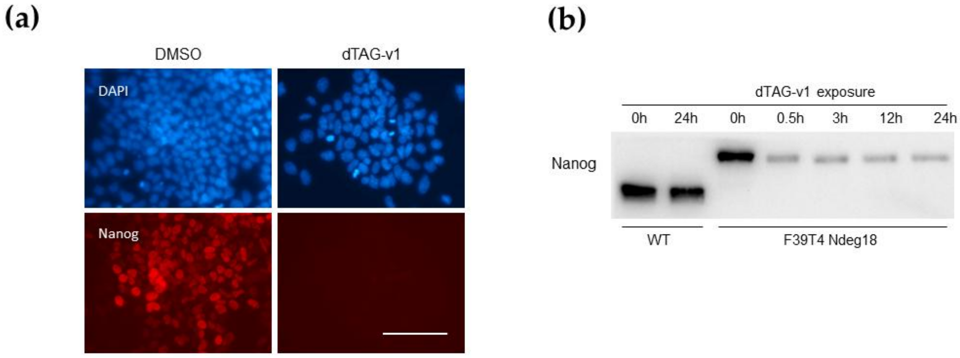

Supplementary Figure S2. dTAG-inducible Nanog degradation in F39T4_Ndeg18 ESC line. (a) Immunocytochemical analysis of Nanog expression following 3 hours of treatment with either DMSO (control) or dTAG-v1 compound. Scale bar, 100 µm. (b) Western blot analysis showing efficient Nanog degradation at different time points following dTAG-v1 treatment.
